# Surgical management of oncologic patient during and after the COVID-19 outbreak: practical recommendations from the Italian society of Surgical Oncology

**DOI:** 10.1007/s13304-020-00921-4

**Published:** 2020-11-12

**Authors:** Davide Cavaliere, Dario Parini, Luigi Marano, Federica Cipriani, Francesco Di Marzo, Antonio Macrì, Domenico D’Ugo, Franco Roviello, Alessandro Gronchi, SICO (Italian Society of Surgical Oncology), Laura Lorenzon, Raffaele De Luca, Ugo Boggi, Guido Torzilli, Secondo Folli, Angelo Restivo, Gaya Spolverato, Alfredo Garofalo, Germana Lissidini, Massimo Dessena, Roberto Girelli, Salvatore Sorrenti, Uberto Fumagalli Romario, Paolo Morgagni, Marco Rastrelli, Ferdinando Cananzi, Maurizio Degiuli, Michele Simone, Annibale Donini, Andrea Muratore, Claudio Belluco, Davide Cavaliere, Dario Parini, Luigi Marano, Federica Cipriani, Antonio Macrì, Domenico D’Ugo, Franco Roviello, Alessandro Gronchi, Giorgio Ercolani

**Affiliations:** 1 General and Oncologic Surgery, Morgagni-Pierantoni Hospital, Ausl Romagna, Forlì, Italy; 2 General Surgery Unit, Santa Maria della Misericordia Hospital, Rovigo, Italy; 3 Department of Medicine, Surgery, and Neurosciences, General Surgery and Surgical Oncology, University of Siena, Viale Bracci 3, 53100 Siena, Italy; 4 Hepatobiliary Surgery Division, Ospedale San Raffaele, Via Olgettina 60, Milano, Italy; 5 UOC Chirurgia Generale, Usl Toscana Sud-Est, Ospedale Valtiberina, Sansepolcro, Arezzo, Italy; 6 Peritoneal Surface Malignancy and Soft Tissue Sarcoma Program, Messina University Medical School Hospital, Messina, Italy; 7 Dipartimento Scienze Mediche e Chirurgiche, UOC di Chirurgia Generale, Fondazione Policlinico Universitario Agostino Gemelli IRCCS, Rome, Italy; 8 Department of Surgery, Fondazione IRCCS Istituto Nazionale dei Tumori, Milan, Italy

**Keywords:** COVID-19, Cancer, Recommendations, Oncologic surgery

## Abstract

The recent outbreak of COVID-19 in Italy caused a limitation of the resources of the health system, which necessarily led to their rationalization in the critical phase (phase 1) and a reorganization of the system in the following phase (phase 2). The Italian Society of Oncological Surgery–SICO has drafted these practical recommendations, calibrated on the most recent scientific literature and taking into account current health regulations and common sense. Surgical activity during phase 1 and 2 should follow a dynamic model, considering architectural structures, hospital mission, organizational models. Surgical delay should not affect oncological prognosis. However, COVID-19-positive cancer patients should be postponed until the infection is cured. The patients to consider more carefully before delaying surgery are those who have completed neoadjuvant therapy, patients with high biological aggressiveness tumors or without therapeutic alternatives. The multidisciplinary discussions are fundamental for sharing clinical decisions; videoconference meetings are preferable and use of telemedicine for follow-up is recommended. Especially in phase 1, maximum effort must be made to reduce the spread of the pandemic. Prefer intra-corporeal rather than open anastomosis during laparoscopy and mechanical rather than hand-sewn anastomosis in open surgery. Consider PPE for caregivers during stoma management. Minimal invasive surgery is not discouraged, because there is little evidence for augmented risk. Specific procedures have to be followed and use of energy devices has to be limited. Training programs with COVID-19 + patients are not recommended. All staff in OR should be trained with specific courses on specific PPE use. Differentiate recommendations are presented for every district cancer. Surgical oncology during phase 2 should be guaranteed by individual and distinct protocols and pathways between cancer patients and COVID-19 + patients with resources specifically addressed to the two distinct kind of patients to limit diagnostic/therapeutic interferences or slowdowns. These recommendations are based on currently available evidence about management of oncologic patients during COVID-19 pandemic, were endorsed by the SICO Executive Board, and are considered suitable for nationwide diffusion. They will be subject to updates and revisions in case of new and relevant scientific acquisitions.

## Background

Dr. Tedros Adhanom Ghebreyesus, Director-General of World Health Organization (WHO), declared the COVID-19 outbreak a public health emergency of pandemic concern on March 11, 2020 [1]. Even though the establishment of lockdown measures by Italian Government to limit the viral spread, today (31/08/20) Italy counts 269.214 confirmed cases including 35.483 deaths, overtaking the total number of infected and deaths so far registered in China [www.salute.gov.it].

Surprisingly, the national health system is proving extraordinarily supportive, responsible and resilient, and many doctors and nurses have been working restlessly since the beginning of the pandemic. It is essential, as well as necessary, to point out that as of 31 August 2020, 177 of our colleagues died and over 29.476 health workers infected (last update 30 June 2020) [dati FNOMCEO—www.portale.fnomceo.it]. The current limited medical resources and the stressed medical staff should be rationally employed to create a high quality and extremely safe environment to protect both patients and health professionals [2].

Moreover, during pandemic Phase 1, the real problem of Italian health system was the lack of enough intensive care facilities, therefore, the number of clinical wards open to patients requiring treatment other than COVID-19 has been reduced, and hospitalizations in these cases have been allowed only in emergency situations or for oncological diseases. As a result, all elective surgical procedures for benign diseases have been postponed to ensure the availability of intensive care facilities [3]. The impact of SARS‐CoV‐2 on elective surgery set a benchmark during the “acute phase” useful to manage the “transition phase” [4].

As indicated in a Multisocietary (surgical and anesthesiologist) Italian document [5], surgical activity during Phase 1 and 2 should follow a dynamic model, considering three main aspects: architectural structures, hospital mission, organizational models. Consequently, the decision to perform or not surgery for cancer patients must be evaluated according to several parameters, both logistical and clinical, and an individual assessment based on the potential detriment of surgery delay on disease progression is mandatory [6] (Table 1).

**Table 1:** Dynamic model for surgical activity during COVID-19 pandemic

Scenario	Census	Resources	Surgical activity
Emergency	> 75% COVID-19-related admissions (ward and ICU)	Significant impact on hospital, healthcare workers and ICU beds Limited ICU and ventilation resources, limited OR resources or a rapid infection increase in the hospital	Emergencies where the patient will not survive unless intervened within the next few hours after a preoperative triage is done by the ethics committee
High level alert	50–75% COVID-19-related admissions (ward and ICU)	Significant impact on hospital, healthcare workers and ICU beds	Emergencies
Medium level alert	25–50% COVID-19-related admissions (ward and ICU)	Impact on hospital resources with pandemic alertness in the hospital with appropriate separate triage in the ER for respiratory symptoms vs non respiratory symptoms ICU beds and wards reserved for COVID-19 patients	Oncologic patients where a lack of treatment would compromise their 3 month’s survival Oncologic patients who cannot receive neoadjuvant treatment to slow progression of disease Oncologic patients who will not require prolonged ICU stay Emergencies
Low level alert	5–25% COVID-19-related admissions (ward and ICU)	No impact on hospital resources but with pandemic alertness in the hospital with appropriate separate triage in the ER for respiratory symptoms vs non respiratory symptoms	Oncologic patients (If an increase in the infection curve is suspected, use “medium level” scenario for oncological surgical activity) Emergencies
Almost normal	< 5% COVID-19-related admissions, without ongoing urgent necessities	No impact on hospital resources	No impact on normal activity

It is well established that cancer-induced immunosuppressive state caused by the malignancy as well as anticancer treatments, such as chemo-radiotherapy or surgery, makes the cancer patients more prone to infections than individuals without neoplasms [7]. Consequently, cancer patients are frail and malnourished and are worthy of attention in the absence of specific guidelines during the outbreak. Chinese surgeons have recently published a proposal for treatment strategy for gastrointestinal tumor under the outbreak of novel coronavirus pneumonia in China [8] and, on the same line, it is imperative to adopt and provide recommendations at national level. With this in view, the Italian Society of Oncological Surgery–SICO has tried to draft these practical recommendations calibrated on the basis of the scientific literature to the best of current knowledge and taking into account current health regulations and common sense.

The present recommendations are based on currently available evidence regarding the surgical and oncological management of the COVID-19 patient, which is, however, lacking and still weak; however, the proposed recommendations were unanimously endorsed by the SICO Executive Board and considered suitable for nationwide diffusion (Table 1). These recommendations will be subject to subsequent updates and revisions in case of new and relevant scientific acquisitions.

## General considerations

As doctors and as surgical oncologists, we should try to find a balance between the need to reduce viral spread, further cancer treatment and optimizing the rational use of healthcare facilities to ensure best clinical practice. However, not all regions of Italy have been equally affected by COVID-19 and it does not seem worthy to mandate the same response univocally.

Given the limited surgical resources, as well as the risks to patients for increased perioperative mortality, whenever possible alternative treatments to surgery could be considered [9]. Patients requiring non-urgent cancer surgery (i.e. in the absence of obstruction, bleeding, perforation symptoms), and COVID-19 positive, should be postponed until the cure of the infection or as the logistical or clinical conditions that caused the postponement change.

For COVID-free cancer patients, the appropriateness of therapeutic strategy should be identified and risk of disease progression should be weighed against the risks of serious postoperative infectious complications, taking into account several parameters [5]; consider an NHS (England) estimation of increased deaths from colorectal cancer of 15–16% due to COVID-19 pandemic [8].

Ideally, COVID-19-negative patients should proceed with the regular surgical pathway COVID-free. As realized in Lombardy, several hospitals should be identified based on their surgical oncology practice and considered as COVID-free. In large hospitals, managing both COVID and no-COVID patients, a COVID-free pathway should be warranted, to decrease the risk of contagion and to protect surgical and medical as well as health care personnel, that can take care of cancer patients [9, 10]. A Hub and Spoke network, above all for high complexity surgical oncological diseases, should be the preferable organizational model [11]. The same model is recommended for Regional and National Oncologic Networks.

### Which oncologic patients should be operated on? At what timing?

**Fig. 1:**
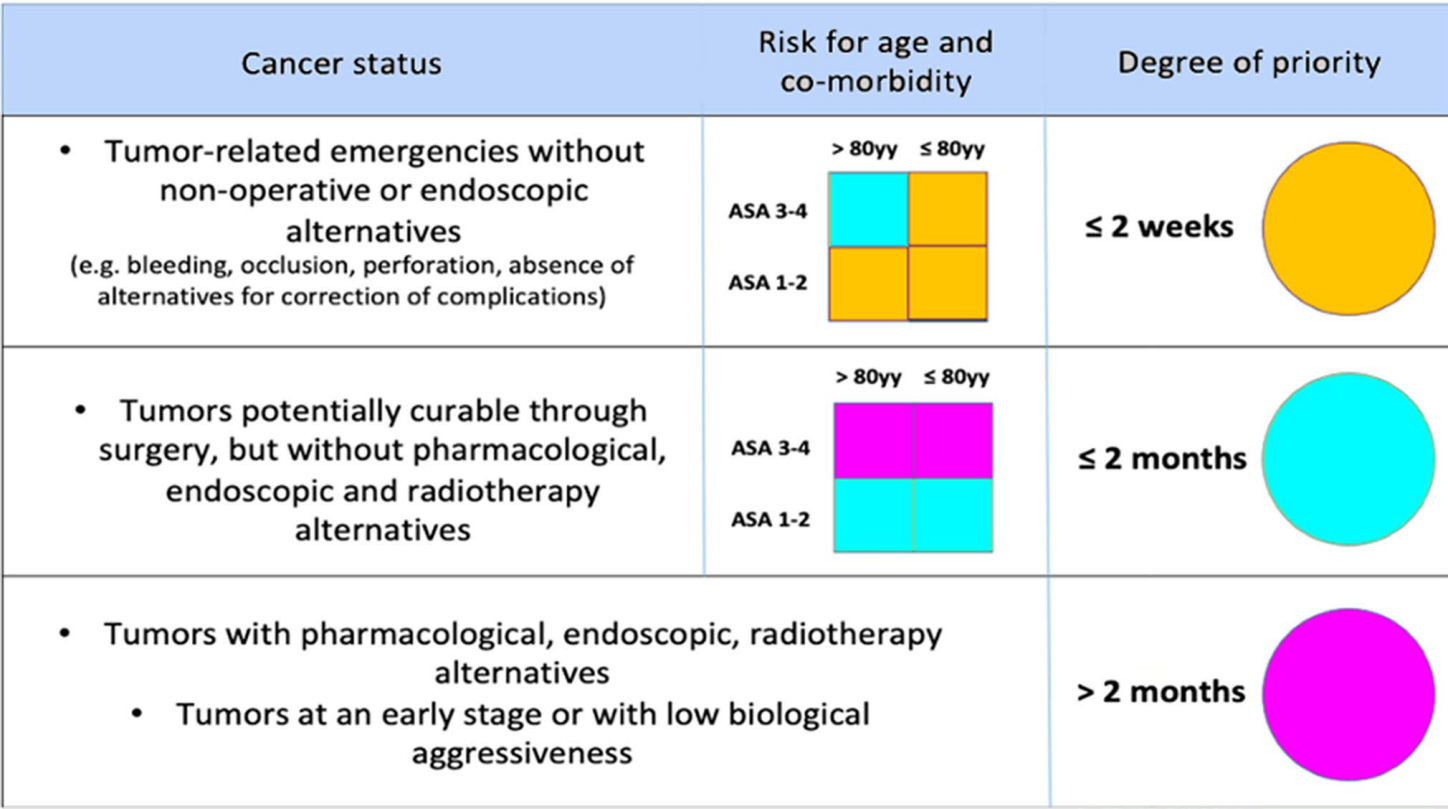
Priority for surgery in COVID-19 free patients with proven abdominal tumors in presence of limited availability of post-operative and intensive care for COVID-19 outbreak

The Regional Health System of Lombardy provides a definition of the different clinical scenarios, described in Fig. 1 [12].

• A careful therapeutic strategy with adequate timing for operative treatment is strongly recommended, which even when the surgery is postponed by the pandemic, should not affect the patient's oncological prognosis; moreover, Italian surgeons should consider the recommendations of the Ministry of Health regarding the waiting list for cancer surgery as indicated in the "National Government Plan for Waiting Lists (PNGLA) 2019–2021" [13].

• The patients to consider more carefully before delaying surgery are: (1) those who have completed neoadjuvant therapy, (2) those affected by neoplasms with high biological aggressiveness, (3) those who do not have therapeutic alternatives.

• Any deviation from the usual treatment scheme and, therefore, also from the priority and timing of cancer treatments should be considered individually on a case-by-case basis by the multidisciplinary group which can meet virtually.

• Waiting lists require periodic review to determine the priority according to the availability of beds and resources.

• The priority of each individual case could change according to the overall situation, tumor biology, clinical response to treatment, age and treatment prospects.

• Referring patients to "COVID-free" or "Less full" Cancer Hubs, where available, should be considered an option in the event of possible delay in the surgical procedure.

### COVID-19 screening before admission

• During pandemic COVID-19, all patients referred to surgery should be considered potentially infected by SARS-CoV-2.

• Prior to admission, screening [14] for SARS-CoV-2 infections should be performed in an outpatient setting or emergency department by means of nurse-directed triage protocol in an external triage tent to stratify the risk of COVID-19, using the following questions: In the last 14 days, have you had fever (> 37.3 °C), cough, sore throat, or respiratory symptoms? Have you had relatives or close contact with a suspicious or confirmed case of COVID-19? Do you come from areas at higher risk of COVID-19?

• Following the preliminary interview, a real-time reverse transcription polymerase chain reaction (rRT-PCR) on respiratory tract specimens should be performed [15]. This exam is actually the gold standard for the etiological diagnosis of SARS-CoV-2 infection [8, 13, 14, 16].

• The same triage protocol should be applied also to patients coming from other health facilities.

• Patients attending elective surgery should all be tested for SARS-CoV-2 infection prior to the planned oncological procedure. In case of positivity, the operation should be postponed until negative viral test, after balancing with the oncological risk.

• Based on the scientific evidence acquired so far, considering the non-specificity of the signs and patterns of high-resolution chest CT-scan (HR-CT) in COVID-19 pneumonia, which do not allow a conclusive diagnosis, HR-CT examination cannot be considered as a substitute for COVID-19 swab, nor used as a means of screening.

### Multidisciplinary tumor board

Telemedicine, in its different forms, has a fundamental role in screening, follow-up and counseling, particularly at this time of social distancing.

As far as the multidisciplinary consultation of the oncological patient is concerned, never as in this period is this a fundamental activity due to the variations in the clinical-assistance pathways compared to the standard imposed by the current crisis situation. However, in case of persistence of social distancing, it is strongly recommended the implementation of videoconferencing platforms to satisfy the requirement of a regular multidisciplinary discussion without delaying the decision-making.

## Specific considerations

Treatment choices that minimize the risk of infection for the health care team should always be considered. In case of procedures with a high potential risk of viral contamination for healthcare workers, it is essential to ensure that adequate personal protective equipment (PPE) is available.

### Bowel anastomosis and stoma creation

• In colorectal cancer surgery, the risk of direct anastomosis, rather than terminal colostomy or protecting it with a lateral ostomy, should be carefully considered due to the specific risk of postoperative complications [8] and, therefore, the need for intensive support at this time lacking, above all in emergency setting.

• Otherwise, when the anastomosis is performed, by laparoscopy is preferable to do an intra-corporeal technique to avoid contamination with aerosolized fecal particles [16]; by laparotomy, mechanical techniques are preferable to hand-sewn, to reduce the risk of contamination by the stool or accidental injury by blades or needles and to shorten operating time.

• Stoma management has to consider as a risk practice for infectious transmission and all caregivers have to pay attention and protect themselves with PPE [14, 17]

### Minimally invasive surgery and infection control

The relative risk of viral infection with exposure to gases during laparoscopy compared to open procedures remains unclear. Conversely, the proven benefits of minimally invasive surgery in terms of reduced hospital stay and postoperative complications are undeniable.

• The first Surgical Societies recommendations were very careful with use of laparoscopy in COVID-19 patients, because the uncertainty about virus presence in aerosol [1, 7, 18].

• Most recent papers, on the other hand [7, 14–16, 19], do not discourage minimally invasive approaches during COVID-19 epidemic; the decision is left to surgeons, who must carefully consider the aspects and risks of their choice.

• The minimally invasive approach is acceptable only if the surgeon is confident with the technique and the operating room staff is well trained in the use and management of specific equipment and technologies, including the safety standards presented below. In general, laparoscopy may reduce intraoperative exposure to aerosolized particles compared with open surgery, but surgeons have to prevent pneumoperitoneum leak through laparoscopic ports or gas evacuation by trocar valves. First of all, skin incisions have to be appropriated to port size, to avoid CO2 spreading; trocars with balloon fixation are suitable [1, 14]. To minimize abdominal pressure effects on respiratory and cardiac functions, pneumoperitoneum has to be as low pressure as possible and time of Trendelenburg position has to be reduced as much as possible [16].

• Just before ending the procedure, whole pneumoperitoneum must be aspirated by the abdominal cavity before removing the trocars or making accessory incision [14].

• During operative time, all staff has to maintain surgical instruments clean from blood and it is mandatory to pay particular attention to avoid sharp injury or gloves and body protection damage [20].

• Throughout the outbreak, it is recommended that all training programs be postponed, including within the institution itself, reserving the execution of minimally invasive procedures for experienced staff who have overcoming the learning curve.

### Surgical smoke, PPE and infection control

Energy-based devices (EBD), including electrocautery, lasers and powered instruments for vessel-sealing or tissue vaporing (e.g. harmonic scalpel, ultrasonic ablator/aspirator and radiofrequency device), are now widely used for surgical dissection and hemostasis both in open and mini-invasive surgery (MIS). Surgical smoke (SS) is defined as the gaseous by-product, visible or microscopic, created by EBD. In the literature, the term SS has various synonyms such as plume, vapors, aerosol. Vital and non-viable cellular materials are included in this SS; therefore, its inhalation is associated with potential health risks. Anyway, the current literature does not confirm the augmented risk for SARS-CoV-2 infection.

### Personal protective equipment during surgery

OR personnel must be trained to recognize, understand, and prevent the risk when managing COVID-19 patients. Surgeons and nurses should be aware of the potential hazards of electrosurgery, and both should communicate openly about dangers in the OR to promote preventive actions.

• All staff should be trained on personal protective equipment (PPE) use: disposable double-layer hats, masks, surgical gowns for single use or medical protective clothing, goggles, or full-face mask, and double-layer sterile gloves [21].

• There is currently no conclusive evidence that SS directly increases the risk of COVID-19 epidemic among OR personnel. However, the high aerosol-infectious potential of SARS-CoV-2 requires a prudent and preventive attitude.

• Although electrocautery is potentially less hazardous than laser or powered energy devices to generate smoke as a route of infection transmission, SS of any kind must be contained.

• The most effective measure to prevent smoking exposure is to limit their use to the mandatory situations. Moreover, protection from SS can be achieved by adoption, possibly combined, of smoke evacuation close to the smoking source (2–5 cm) in open surgery, or on trocars luer-lok valves during pneumoperitoneum, using special filters that allow continuous ventilation and filtration of the pneumoperitoneum, filtered central wall room suction units, and the use of personal filtration masks to prevent the inhalation of particles and any infectious biological agents.

• There is no agreement too on the type of respiratory protection. A major concern is that standard surgical masks often fit loosely and allow the inhalation of aerosolized particulate matter, bypassing filtering. A standard surgical mask protects from aerosolized particles greater than 5 μm in size. Bacterial and viral particles from 0.04 to 1.3 μm spread easily through surgical masks, with the result that these masks do not meet the minimum respiratory protection standards required by the Occupational Safety and Health Administration [22]. Instead, higher performance masks (e.g. FFP2, N95 respirator or equivalent) are very effective and protect against a higher number of microscopic elements and has been shown to provide better protection against such aerosolized infectious pathogens [23]; at least carefully fit-tested double masks can increase filtration capacity and are therefore recommended.

• According to the Association for the Advancement of Medical Instrumentation [21], the protection level of the surgical gowns depends on the type of procedure. The highest levels (3 and 4) are the highest and moderate fluid and microbial barrier needed for long, fluid-intensive procedure.

## Specificity by cancer type

### Breast

High-grade invasive cancers (≥ T2, G3, high Ki67 levels, triple negative HER2 + , N1, inflammatory carcinoma) should be considered for neoadjuvant treatment. We propose these priority access for upfront surgery:

• High priority (surgery within 30 days from diagnosis):

- High-grade invasive tumors (G3, high ki67 levels, triple negative HER2 + , N1) in premenopausal women without indications for neoadjuvant treatments.

- Non-responders or in progression patients during neoadjuvant treatments.

- Pregnant women.

- Invasive T2 tumors (> 3 cm) with no indications for neoadjuvant treatment.

- Isolated loco-regional recurrence within 48 months from the primary event.

- Ulcerated as well as bleeding tumors.

• Medium priority (surgery within 60 days from diagnosis):

- After the neoadjuvant treatment.

- cT1, n0, luminal A.

• Low priority (surgery within 90 days from diagnosis):

- Luminal A cancers in post-menopausal women.

- In situ carcinomas.

### Esophagus

• Neoplasms limited to submucosal layer: operative treatment, both surgical and endoscopic, should be postponed for at least 2 weeks, or until the epidemic is controlled.

• Stage II–III: recommend neoadjuvant treatment before surgery.

• The neoadjuvant treatment should be prolonged in patients with good tumor response as well as high tolerability, aiming to postpone the surgical approach.

• Emergency/urgency setting (fistula, haemorrhage, obstruction): consider endoscopy and/or interventional radiology as a first approach and the same applies to the management of post-operative complications.

### Stomach

• Neoplasms limited to submucosal layer: operative treatment, both surgical and endoscopic, should be postponed for at least 2 weeks, or until the epidemic is controlled.

• Early gastric cancers or non-locally advanced malignancies (≤ cT2N0): since gastric cancer is considered neoplasm with high biological aggressiveness, in these early cases, the goal of radical surgery with curative intent should be obtained.

• More advanced cancers (> cT2N0): neoadjuvant chemotherapy/radiotherapy should also be recommended and re-evaluation for radical surgery after the epidemic control should be scheduled.

• Post-neoadjuvant chemotherapy patients can continue the medical treatment if responding and tolerating it.

### Colon–rectum

• Elective treatment, both endoscopic and surgical for malignant polyps should be postponed until control of epidemic.

• For invasive non-metastatic colon cancers (cT ≥ 3 N +) should be considered the neoadjuvant treatment.

• For stage II–III rectal cancers, the systemic neoadjuvant chemotherapy should be considered.

• Post-neoadjuvant chemotherapy patients can continue the medical treatment if responding and tolerating it, after multidisciplinary evaluation, if the surgical treatment cannot be planned in scheduled time.

• For obstructive colon cancers (with clinical symptoms or with pre-obstructive signs at imaging), the resection of primary tumor should be carried out.

• For obstructive rectal (extraperitoneal) cancers (with clinical symptoms or with pre-obstructive signs at imaging), the derivative colostomy should be recommended.

### Hepato-pancreato-biliary cancers

Patients with marginally resectable hepato-pancreato-biliary tumors, with no other therapeutic alternatives, and with aggressive behavior, should undergo upfront surgery with minimal variation of scheduling time. For all other patients, if surgery cannot be offered within the scheduled time, the start or continuation of neoadjuvant chemotherapies, chemoembolization, ablation, radioembolization should be considered as a possible treatment also to defer surgery, when appropriate.

• Hepato-biliary cancers.

- In patients with hepatocarcinoma (HCC) is recommended to consider chemoembolization, ablation, radioembolization as treatment options to bridge with surgery at the end of outbreak.

- In cholangiocarcinoma patients, since the limited efficacy of alternative therapies, upfront surgery should not be postponed.

- Only patients with high MELD or HCC should be evaluated for liver transplant.

• Pancreas cancers.

- In pancreas adenocarcinoma, neoadjuvant treatments are recommended.

### Peritoneal malignancies

• In case of suspected peritoneal malignancy with low diagnostic susceptibility at common diagnostic tools, diagnostic laparoscopy should be performed within 2–4 weeks.

• In case of histologically proven low-grade peritoneal malignancy, without symptoms, the treatment could be postponed of 2–4 months.

• For high-grade malignancies the neoadjuvant therapy should be performed.

• After neoadjuvant treatment, surgery should be scheduled within 4–6 weeks.

• PIPAC should not be postponed, since the unfavorable prognosis of patients and not requiring the intensive care unit recovery.

• The HIPEC as well as PIPAC produce high levels of aerosol. Therefore, all OR staff must wear the PPE at maximum level of protection.

### Endocrine system

Surgical resection should not be postponed in the following cases:

• Thyroid: Tir4 and Tir5 nodules with infiltrative aspects of capsula as well as surrounding tissues and/or organs, or with metastatic lymph nodes. Additionally, patients with distant metastases and patients submitted to lobectomy/isthmectomy needing total thyroidectomy, not to delay the radiometabolic treatment.

• Parathyroid: in cases with serum calcium values more than 12 mg/dl or with suspected parathyroid carcinoma infiltrating the thyroid gland or surrounding tissues.

• Adrenal gland: in cases with significant metabolic syndromes or with suspected malignancy at imaging.

### Soft tissue sarcomas

• A primary soft tissue sarcoma without metastatic disease on staging that needs surgery will be prioritized for the OR.

• Deferring the surgical treatment of newly diagnosed truncal/extremity well-differentiated liposarcoma/ALT, classic dermatofibrosarcoma protuberans and desmoids for at least 3 months or more. Will reassess at that time.

• Resection of other low-grade lesions with known indolent behavior (e.g., retroperitoneal well-differentiated liposarcoma) and low metastatic risk (e.g., myxoid liposarcoma, low-grade fibromyxoid tumor) can be deferred for short intervals depending on available resources and presence/absence of systemic symptoms.

• Consider deferral of re-excision for R1 margins in extremity/truncal lesions if OR resources are limited and there is no evidence of residual disease on post unplanned excision assessment.

• If there is an indication for radiation therapy, will plan to do it preoperatively. This can be administered in a lower risk outpatient setting and will push out the timing of surgery for about 3–4 months. In addition, consider the use of preoperative RT as a bridge therapy to postpone surgery when appropriate, even if the treatment is not standard, but there is evidence that it will not harm (i.e. preoperative RT in retroperitoneal liposarcoma).

• Use of neoadjuvant therapy for high-risk sarcomas at any site or recurrent disease can be considered if it can be safely delivered in an outpatient setting as a means of deferring surgical intervention.

• Use neoadjuvant imatinib in localized GIST as a bridge therapy even if a formal indication to neoadjuvant therapy does not exist on clinical grounds, providing the mutation is sensitive.

• Active observation protocols or low-toxicity systemic options can be considered for patients with recurrent disease. Surgery for recurrent disease can be offered to patients who: are likely to have relatively high chances of obtaining long-term disease control in the context of complete gross resection (e.g., long disease-free interval, solitary site of recurrence) require immediate palliation (e.g., due to bleeding, obstruction) and who do not have indolent histologies (e.g., well-differentiated liposarcoma in the retroperitoneum or classic solitary fibrous tumor) that can be managed with active observation.
